# Bioprospecting of powdered pineapple rind as an organic supplement of composted sawdust for *Pleurotus ostreatus* mushroom cultivation

**DOI:** 10.1002/fsn3.551

**Published:** 2017-11-29

**Authors:** Deborah L. Narh Mensah, Peter Addo, Matilda Dzomeku, Mary Obodai

**Affiliations:** ^1^ CSIR – Food Research Institute Accra Ghana

**Keywords:** biological efficiency, fruiting body, nutritional composition, yield

## Abstract

Pineapple rind is a by‐product of the pineapple processing industry and contains nutrients and other compounds which must be utilized as a bioresource for socio‐economic benefits while preventing the potential problems of improper agroindustrial biomass disposal methods. *Pleurotus ostreatus* is an edible oyster mushroom with medicinal properties and can be cultivated on various agroindustrial biomass, including sawdust containing supplements. Pineapple rind was powdered and used as a supplement of composted sawdust at 2%, 5%, 10%, 12%, 15%, and 20% (w/w) on dry weight basis. A control treatment consisted of composted sawdust supplemented with rice bran at 12% (the most utilized composition in Ghana). *P. ostreatus* strain EM‐1 was cultivated on these treatments. Factors investigated included the spawn run period, yield, fruiting body weight and size, biological efficiency, and nutritional composition (proximate composition and Copper, Zinc and Lead content) of fruiting bodies harvested from selected high‐yielding treatments and the control treatment. Full colonization of all treatments occurred by the 34th day of incubation. Enhanced yield, fruiting body weight and size, and biological efficiency were generally recorded with supplementation at lower concentrations (2% and 5%) compared to treatments supplemented at higher concentrations. There was also a supplement concentration‐dependent alteration of the nutritional composition of the mushroom. Powdered pineapple rind can be utilized as an organic supplement at relatively low concentrations in composted sawdust for *P. ostreatus* strain EM‐1 cultivation. The use of lower concentrations of powdered pineapple rind in composted sawdust is advantageous as relatively less input will be required to produce higher *P. ostreatus* strain EM‐1 yields. Utilization of pineapple rind for mushroom cultivation will extend the pineapple plant value chain, intensify mushroom production in a sustainable way, and minimize agricultural losses.

## 1 INTRODUCTION

Ghana's horticultural export market expanded extensively over a period of 10 years with pineapple (*Ananas comosus*) championing this market. Pineapple is mostly consumed fresh within the country, but processed products including packaged fresh‐cut pineapple, cut‐dried pineapple, packaged fruit salads, commercially juiced, and bottled pineapple, and juicing in individual households are also available (Danielou & Ravry, 2005). Pineapple rinds and cores form about 40% of the whole fruit (Ketnawa, Sai‐Ut, Theppakorn, Chaiwut, & Rawdkuen, 2009) but are generally not utilized. Because of this, while generating a source of income for stakeholders, the pineapple processing industry is prone to creating a large mass of by‐products from the parts of the fruit that are not eaten (rinds and cores), which could contribute to environmental pollution and its associated problems if not well managed. The management of this material should not only be geared toward disposal methods, but rather to the utilization of this material as a bioresource for socioeconomic benefits.

The contents of the material should be considered in identifying suitable uses of the resource. Pineapple rinds have been reported to contain considerable amount of soluble sugars, as well as high fiber, relatively low protein content, vitamins, and carotenoids such as β‐carotene and lutein. Although pineapple rinds contain minimal levels of bromelain, a proteolytic enzyme, the enzyme could be inactivated with thermal processing (Freitas et al., 2015; Ketnawa, Sai‐Ut, Theppakorn, Chaiwut, & Rawdkuen, 2009; Sriwatanapongse, Balaban, & Teixeira, 2000; Umesh Hebbar, Sumana, & Raghavarao, 2008). Bioconversion of pineapple rinds could be used to produce several beneficial bioactive compounds and products including methane, ethanol, bromelain, and sources of single‐cell proteins (Correia et al., 2004).

*Pleurotus ostreatus* is a nutritious mushroom species with some medicinal properties (Mattila et al., 2001; Obodai, Ferreira, et al., 2014; Reis, Barros, Martins, & Ferreira, 2012) and has been the most widely cultivated mushroom since the inception of commercial mushroom cultivation in Ghana, with sawdust serving as the main substrate (Obodai & Johnson, 2002). Efforts have been made to investigate other substrates singly and in various combinations and pretreatments to establish their suitability for mushroom cultivation in the country. Among these investigations is the research into utilization of rice straw given various pretreatments with or without the addition of sawdust of *Triplochiton scleroxylon* and supplementation with rice bran as reported by Obodai, Narh, Baka, and Dzomeku (2011). Sawdust is presently supplemented with rice bran or wheat bran for *Pleurotus ostreatus* strain EM‐1 production although the utilization of rice husk and fish waste as a supplement for cultivation of the mushroom has also been evaluated by Frimpong–Manso, Obodai, Dzomeku, and Apetorgbor (2011) and Ottah‐Atikpo et al. (2007) in the country. Other organic supplements used in addition to rice bran in other countries include blood meal, fish meal, cotton seed meal, chicken manure, brewer grain, malt sprouts, and corn bran (Oei, 2016).

This study seeks to investigate the suitability of pineapple rind as an organic supplement of composted sawdust in the cultivation of *P. ostreatus* in an effort to minimize the risk of the previously described potential problems of improper pineapple waste disposal methods. It is also expected that utilization of powdered pineapple rind for *P. ostreatus* cultivation and the consumption of mushrooms produced using this substrate will extend the pineapple plant value chain while contributing to poverty reduction and food and nutrition security.

## 2 MATERIALS AND METHODS

### 2.1 Spawn and compost preparation

Cultures of *P. ostreatus* (Jacq.ex.Fr.) Kummer strain EM‐1 originally from Mauritius and maintained on Potato Dextrose Agar slants were used to prepare spawn. Spawn of this mushroom was prepared, using grains of sorghum and millet, obtained from the Nima Market in Accra, combined in a 3:1 (w/w) ratio (Narh, Obodai, Baka, & Dzomeku, 2011). Compost was prepared by the outdoor single‐phase solid waste fermentation method as described by Obodai and Johnson (2002).

### 2.2 Powdered pineapple rind preparation

Rind from pineapples (*Ananas comosus var*.MD2) was collected from Nature's Best fruit processing incubation factory at CSIR – Food Research Institute, and solar dried for 45 hr to a moisture level of about 18% as determined, using the AOAC method (AOAC, 1990). The dried rind was milled using a domestic blender/mill (Mammonlex blender/mill model ms‐223 Taiwan). The powder obtained was referred to as Powdered Pineapple Rind (PPR). The PPR was stored in dark polyethylene bags until use.

### 2.3 Treatment preparation and mushroom cultivation

PPR was added to the compost at bagging to final concentrations of 2, 5, 10, 12, 15, and 20% (w/w) on dry weight basis before bagging. A control treatment supplemented with rice bran (RB) at 12% (w/w) was also set up. Aliquots of quicklime were added to all treatments at 0.5% (w/w). The moisture content of each treatment was also adjusted with tap water to approximately 60%–70% using the squeeze test (Buswell, 1984). The mixtures were bagged, sterilized, and inoculated with approximately one table spoon full (about 5 g) of *P. ostreatus* (Jacq.ex.fr) Kummer strain EM‐1 spawn, incubated till total spawn run and cropped as previously described by Obodai and Johnson (2002). The treatments were labeled 2% PPR, 5% PPR, 10% PPR, 12% PPR, 15% PPR, 20% PPR, and 12% RB for bags supplemented with PPR at 2%, 5%, 10%, 12%, 15%, and 20% PPR, and RB at 12%, respectively. Following sterilization, the acidity of the treatments was measured as described by Narh, Obodai, Baka, & Dzomeku (2011) with some modification. In brief, 5 g of each treatment was separately steeped in 100 ml of distilled water for 20 min. The pH was taken at 25°C with a pHM92 Laboratory pH meter (MeterLabTM, Radiometer Analytical A/S, Copenhagen, Denmark). There were 5 replicates per treatment.

Data collected during incubation included the mycelia growth rate and the spawn run period (days till total colonization).

Fresh mushrooms were harvested at maturity during cropping. Data collected during the cropping stage included weight and number of fruiting bodies per flush, weight and number of fruiting bodies per bag, and the total yields per bag. Physical characteristics of the fruiting bodies recorded included fruiting body weight, cap and stipe weights, cap diameter and stipe circumference of five randomly selected fruiting bodies of the first flush of mushrooms. Caps and stipes of the selected fruiting bodies were separated by cutting the stipe at the cap attachment with a scalpel. Weights were taken with a Digital Computing Scale (Hana Electronics Company Limited, Korea). Cap diameters were taken by measuring the diameter of the cap at right angles for each randomly selected fruiting body and averaging the values to represent the cap diameter for that fruiting body. An average of the circumferences of the largest and smallest portion of the stipe was recorded as the stipe circumference of each selected fruiting body.

The ratio of cap weight to stipe weight was determined by dividing the average cap weight of the five randomly selected fruiting bodies by the average stipe weight of the same fruiting bodies. Ratio of cap diameter to stipe circumference was also determined by dividing the average cap diameter of the same five randomly selected fruiting bodies by the average stipe circumference of the five fruiting bodies. The total yield per flush as well as the total number of fruiting bodies were also recorded. Mushroom size was the ratio of mean total weight of fresh mushroom harvested per bag and the mean total number of mushrooms harvested per bag as described by Royse, Rhodes, Ohga, and Sanchez (2004). The biological efficiency (BE) was determined as the percentage of total weight of fresh mushrooms to dry weight of substrate at spawning as described by Royse, Rhodes, Ohga, and Sanchez (2004).

### 2.4 Determination of nutritional composition

The entire fruiting bodies (cap and stipes from all flushes) from treatments showing the highest biological efficiencies as well as the control treatment were separately sun dried, milled, and kept refrigerated at −10°C until chemical analyses were conducted.

#### 2.4.1 Proximate composition

Nutritional composition (moisture, ash, fat, protein, carbohydrates, and energy) of the milled samples were determined, using the AOAC procedures (AOAC, 1990, 2000). Sample crude protein content (*N* × 4.38) was estimated by the Macro‐Kjeldahl method. The crude fat was determined by extracting a known weight of sample with petroleum ether, using a Soxhlet apparatus. Ash content was determined by incineration at 600 ± 15°C for 24 hr. The total carbohydrates were calculated by difference whereas the total energy was calculated according to the equation,

Energy (kcal)=4×(g protein+g carbohydrates)+9×(g fat).

#### 2.4.2 Mineral contents

All the minerals; Copper (Cu), Zinc (Zn), and Lead (Pb) were analyzed by AAS (Buck Scientific 210VGP Flame AAS, Buck Scientific, Inc. East Norwalk, USA) after sample wet digestion according to the method described by the authors (Obodai, Ofori, et al., 2014).

### 2.5 Data collection and statistical analysis

With the exception of the nutritional composition, which was determined in replicates of the total yields obtained from respective treatments, all other analyses were conducted with five randomly selected replicates. Means and standard deviation or standard errors were calculated in Microsoft Excel 2013.

## 3 RESULTS AND DISCUSSION

### 3.1 pH and moisture content of treatments and mycelial growth rate of *P. ostreatus* strain EM‐1 on treatments

The pH of all the treatments, including the control ranged from 6.2 to 7.7. These values are generally within the optimal pH range (6.0–6.8) reported (Stamets, 2000) for *P. ostreatus* cultivation. Moisture content of all the treatments was relatively lower than the optimal moisture content required for *P. ostreatus* cultivation (85%–95%) (Stamets, 2000). Moisture content of the substrates ranged from 60 to 70%.

**Figure 1:**
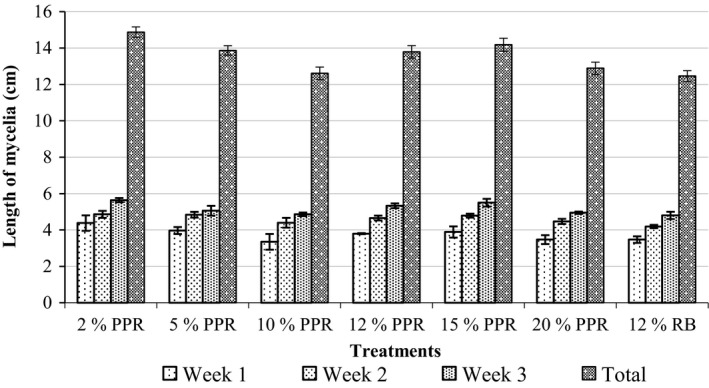
Radial mycelia extension per treatment. Total length of mycelia refers to length of mycelia by the end of the third week of incubation. Error bars are standard errors. *n* = 5

The highest mycelia extension for all treatments were observed in Week 3 of incubation and lowest in Week 1 (Figure 1). The increasing order of mean mycelia lengths was 12.5, 12.6, 12.9, 13.8, 13.9, 14.2, and 14.9 cm for treatments 12% RB, 10% PPR, 20% PPR, 12% PPR, 5% PPR, 15% PPR, and 2% PPR, respectively, by the end of the third week of incubation. The mycelia length of treatment 10% PPR was comparable to that of the control treatment, whereas treatment 2% PPR recorded the significantly highest mean mycelia length (*p* < .05) by the end of the third week. Spawn run periods for the treatments with varying concentrations of pineapple rind were comparable to the control treatment and differed by 1 or 2 days (Table 1). All treatments were fully colonized by the 34th day of incubation (Table 1).

**Table 1:** Days till total colonization, total yield, total number of fruiting bodies per bag, mushroom size, and biological efficiency of *P. ostreatus* on treatments within the cropping period

Treatment	Days till total colonization (days)	Total yield per bag (g)	Total number of fruiting bodies per bag	Mushroom size (g/fruiting body)	BE (%)
2% PPR	31 ± 1a	236.5 ± 37.00bc	25 ± 3b	9.42 ± 0.61b	78.83 ± 12.33bc
5% PPR	33 ± 1a	223.5 ± 14.95c	23 ± 2b	10.00 ± 0.66b	74.50 ± 4.98c
10% PPR	33 ± 1a	163.4 ± 14.74a	17 ± 2a	5.32 ± 0.79a	54.47 ± 4.91a
12% PPR	32 ± 1a	186.7 ± 23.97ab	20 ± 2ab	5.59 ± 1.31a	62.23 ± 7.99abc
15% PPR	32 ± 1a	195.7 ± 16.99ab	24 ± 4b	9.72 ± 1.28b	65.23 ± 5.66b
20% PPR	33 ± 1a	193.36 ± 32.42abc	23 ± 3b	8.20 ± 0.72b	64.45 ± 10.81ab
12% RB (Control)	33 ± 1a	195.7 ± 10.21b	21 ± 1b	9.60 ± 0.72b	65.23 ± 3.40b

Values presented are means ± standard error. *n* = 5. Values in each column followed by different letters (“a” to “b” or “c”, “a” being the lowest value) mean statistically significant differences between samples (*p *<* *.05).

Growth of *P. ostreatus* strain EM‐1 on rice husk as additive on *T*. *scleroxylon* revealed some treatments exhibited relatively longer spawn run periods, extending up to 47 days (Frimpong–Manso et al., 2011). Short spawn run periods are ideally desirable for commercial cultivation of mushrooms as this would minimize both the cultivation cost and the time required for accruing returns. Short spawn run periods would also increase the number of cultivation cycles per growing room. Hence, sustainably enhance *P. ostreatus* strain EM‐1 cultivation with a resulting increase in profit per growing facility. However, Royse (1985) reported that longer spawn run periods of *Lentinula edodes* cultivated on sawdust substrate supplemented with wheat and/or millet bran resulted in 2 or 3 times higher biological efficiencies and larger mushrooms than shorter spawn run period. In our opinion, short spawn run periods are beneficial so far as the mycelia fully colonize the substrate within the incubation time. These results depict the dependence of mycelia extension on the substrate formulation in terms of the type and proportion of supplement added, and corroborate the findings of Royse, Rhodes, Ohga, and Sanchez (2004) regarding *P. cornucopiae*.

### 3.2 Mushroom yield parameters

**Figure 2:**
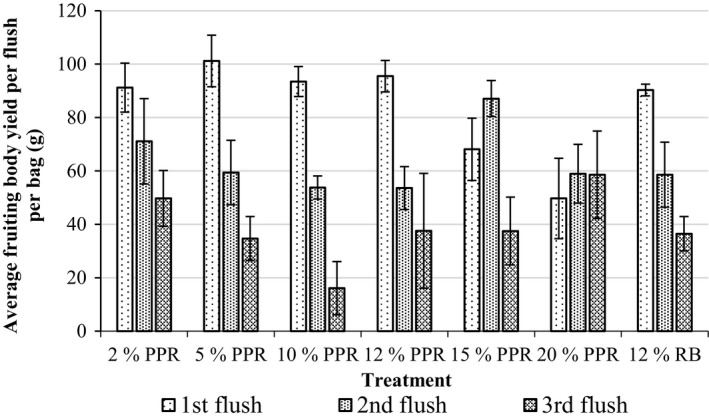
Fruiting body yield per flush per bag. Error bars are standard errors. *n* = 5

The yield per flush per bag for each treatment is presented in Figure 2. Yields obtained were generally highest in the first flush and reduced with subsequent flushes. This trend in flushing has been severally reported (Mshandete & Cuff, 2008; Obodai, Narh, Baka, and Dzomeku (2011); Tisdale, Miyasaka, & Hemmes, 2005) for oyster mushrooms, including the strain presently studied, and other species of mushrooms.

Days till total colonization, total yield, total number of fruiting bodies per bag, mushroom size, and BE of *P. ostreatus* on treatments within the cropping period are presented in Table 1.

Pineapple rind at 2% and 5% supplementation (2% PPR and 5% PPR) both supported the best yield of the fungus in comparison with all the other treatments supplemented with PPR. However, yields obtained from 2% PPR treatment (236.5 ± 37.00) were somewhat comparable to that from the 5% PPR (223.5 ± 14.95) and the control treatment (12% RB; 195.7 ± 10.21). The yields obtained from 5% PPR were statistically higher (*p* < .05) than that of the control treatment.

Although Thomas, Prabhu, Reeny, and Bopaiah (1998) have reported that the yield of the mushroom is directly related to the spread of mycelium into the substrate, our results demonstrate that the trends for mycelia extension and fruiting body yield differ. This indicates that mycelial growth and yield of mushrooms have different requirements as has been documented by Oei (1996).

The mean total number of fruiting bodies obtained per bag during the cropping period ranged from 17 to 25. The mushroom size of the 2% and 5% PPR treatments (the highest yielding treatments containing PPR) were statistically not significantly different (*p* > .05) from that of the fruiting bodies obtained from the control treatment (12% RB). As observed for the yields, the highest BE was recorded for the 2% PPR treatment (78.83 ± 12.33%), which was comparable to the BEs of the 5% PPR treatment (74.50 ± 4.98%) and the control treatment (65.23 ± 3.40%). Formulation of sawdust and other substrates have been previously reported (Frimpong–Manso et al., 2011; Mamiro & Mamiro, 2011; Royse et al., 2004) to influence yield, mushroom size, and biological efficiencies of the main substrate on various species and/or strains of mushrooms because of the nutrients available to the mushroom mycelia in the formulated substrate. Moreover, other studies (Frimpong–Manso et al., 2011; Obodai & Johnson, 2002) carried out in Ghana showed that rice husk at 2% supplementation gave an increase in yield of 11% over the control whereas groundnut testa at 15% supplementation gave an overall increase in yield of 57% over the control treatment. This study demonstrates that supplementation of composted sawdust with PPR influences the yield and mushroom size of *P. ostreatus* strain EM‐1 and the BE of composted sawdust for *P. ostreatus* strain EM‐1 cultivation. However, PPR improves these parameters in *P. ostreatus* strain EM‐1 when used as a supplement in composted sawdust at relatively lower concentrations.

### 3.3 Weights and dimensions of fruiting bodies harvested in the first flush

Whole fruiting body, cap, and stipe weights, cap diameter, and stipe circumference of five fruiting bodies from the first flush of mushroom are presented in Table 2. Variations were generally observed for all the parameters measured for the various treatments with average fruiting body weights of 6.9 g for treatment 20% PPR and 13.0 g for treatment 2% PPR. With the exception of treatments 15% PPR and 20% PPR, which had significantly lower cap weights compared to the control treatment (12% RB), the cap weights of all treatments supplemented with PPR at 2%–12% supplementation were comparable with the control treatment. Cap weights were significantly lower (*p* < .05) in treatments containing relatively high concentrations of PPR (15 and 20%) compared to all the other treatments, including the control treatment. Stipe weights followed similar trends and ranged from a mean weight of 1.0 to 6.0 g across the treatments. Mean cap diameters ranged from 5.3 cm to 7.0 cm whereas mean stipe circumferences ranged from 2.4 cm to 3.2 cm across the treatments. The increasing order of the ratio of cap weight to stipe weight was 1.3, 3.0, 3.1, 4.1, 5.7, 5.8, and 5.9 for treatments 5% PPR, 10% PPR, 2% PPR, 12% PPR, 12% RB, 20% PPR, and 15% PPR, respectively. The dimensional ratio of the cap to the stipe ranged from 2.0 to 2.4 across the treatments.

**Table 2:** Fruiting body weight and size parameters of first flush

Treatment	Fruiting body weight (g)	Cap weight (g)	Stipe weight (g)	Cap diameter (cm)	Stipe circumference (cm)	Cap to stipe weight ratio	Cap to stipe dimensional ratio
2% PPR	13.0 ± 1.01b	9.7 ± 0.71b	3.1 ± 0.30c	7.0 ± 0.22b	3.0 ± 0.19ab	3.1:1	2.3:1
5% PPR	12.3 ± 1.43b	7.8 ± 1.84ab	6.0 ± 3.12c	6.6 ± 0.21ab	2.8 ± 0.07a	1.3:1	2.3:1
10% PPR	11.2 ± 2.19b	8.4 ± 1.58ab	2.8 ± 0.64c	5.9 ± 1.22ab	2.4 ± 0.50a	3.0:1	2.4:1
12% PPR	11.9 ± 2.36b	9.5 ± 1.92ab	2.3 ± 0.49bc	6.7 ± 0.79ab	3.0 ± 0.22ab	4.1:1	2.3:1
15% PPR	7.7 ± 1.78ab	6.6 ± 1.57a	1.1 ± 0.21a	6.2 ± 0.59a	3.2 ± 0.42ab	5.9:1	2.0:1
20% PPR	6.9 ± 1.90a	5.9 ± 1.64a	1.0 ± 0.25a	5.3 ± 0.81a	2.7 ± 0.56ab	5.8:1	2.0:1
12% RB (Control)	12.0 ± 0.85b	10.0 ± 0.83b	1.8 ± 0.21b	6.8 ± 0.33b	3.0 ± 0.17b	5.7:1	2.3:1

Values presented are mean±standard error. *n* = 5. Values in each column followed by different letters (“a” to “b” or “c”, “a” being the lowest value) are significantly different (*p *<* *.05).

The fruiting body parameters reported herein are comparable to that reported by Baka, Narh, Obodai, Dzomeku, and Takli (2010) for the same strain of *P. ostreatus* cultivated on composted sawdust and cropped using a variety of cropping techniques. It is, however, noteworthy that supplementation of composted sawdust with PPR at relatively high concentrations (≥15% w/w) has a negative influence on the cap and stipe weights as well as the cap diameters of fruiting bodies obtained from the substrate.

### 3.4 Nutritional composition of fruiting bodies

Moisture, ash, fat, protein, carbohydrate, and energetic values of the powdered mushroom fruiting bodies ranged from 10.56 to 10.84 g/100 g.dw, 8.34 to 8.40 g/100 g.dw, 1.29 to 1.45 g/100 g.dw, 20.4 to 21.65 g/100 g.dw, 57.85 to 59.08 g/100 g.dw and 329.5 to 334.34 Kcal/100 g.dw, respectively (Table 3). The ash content of the samples statistically did not differ significantly (*p* > .05), whereas there were statistically significant differences (*p* < .05) in the values for the other nutritional parameters. For instance, the significantly highest and lowest protein contents were recorded in the fruiting bodies obtained from treatments 2% PPR and 5% PPR respectively. Significantly lower values of copper, zinc, and lead were recorded for fruiting bodies obtained from treatment 2% PPR while the values recorded for the fruiting bodies obtained from treatments 5% PPR and 12% RB did not differ significantly.

**Table 3:** Proximate composition, microelements, and heavy metal content of fruiting bodies from highest yielding treatments and control treatment

Component	2% PPR	5% PPR	12% RB (control)
Proximate composition			
Moisture	10.69 ± 0.02b	10.84 ± 0.04c	10.56 ± 0.12a
Ash	8.38 ± 0.13a	8.40 ± 0.09a	8.34 ± 0.05a
Fat	1.45 ± 0.06b	1.29 ± 0.06a	1.98 ± 0.18c
Protein	21.65 ± 0.05c	20.41 ± 0.01a	21.04 ± 0.04b
Carbohydrates	57.85 ± 0.16a	59.08 ± 0.02c	58.10 ± 0.05b
Energy	331.01 ± 0.34b	329.51 ± 0.54a	334.34 ± 1.60c
Microelements			
Copper	0.19 ± 0.01a	0.43 ± 0.01b	0.40 ± 0.04b
Zinc	0.63 ± 0.35a	1.29 ± 0.23b	1.15 ± 0.04b
Heavy metal			
Lead	1.20 ± 0.33a	1.89 ± 0.15b	1.89 ± 0.13b

Values presented are mean ± standard deviation. *n* = 2. In each row, different letters (“a” to “b” or “c”, “a” being the lowest value) mean statistically significant differences between samples (*p* < .05). With the exception of energetic value reported in Kcal/100 g.dw, the nutritional values are reported in g/100 g.dw. Microelements and heavy metal are reported in mg/100 g.dw.

Ash, carbohydrate, copper, zinc, and lead contents of this study were relatively higher while all the other nutritional values were relatively lower than that reported by the authors (Obodai, Ferreira, et al., 2014) for the same strain of *P. ostreatus* cultivated on composted sawdust. Consumption of *P. ostreatus* and other mushrooms is usually based on the nutritional composition, medicinal value, and organoleptic properties, which sometimes differ in the caps and stipes of the fruiting bodies (Oboh & Shodehinde, 2009).

These results demonstrate that substrate formulation influences the nutritional and mineral composition of fruiting bodies of *P. ostreatus*. In addition, the concentration of PPR in composted sawdust is relevant for altering the nutritional characteristics of the mushroom's fruiting bodies obtained from composted sawdust.

## 4 CONCLUSIONS

This study shows that supplementation of composted sawdust with PPR at low concentrations results in yields and mushroom size of *P. ostreatus* strain EM‐1 comparable to the mushroom cultivated on composted sawdust supplemented with rice bran at 12%. PPR at low concentrations also significantly enhances the biological efficiency of composted sawdust in comparison with supplementation with rice bran at 12%. The study also demonstrates the supplement concentration‐dependent alteration of the nutritional composition of the mushroom. Therefore, PPR can be considered to be a good organic supplement of composted sawdust for *P. ostreatus* strain EM‐1 cultivation when used at low concentrations.

## CONFLICTS OF INTEREST

The authors declare that they have no known conflict of interest.
